# Targeted Killing of Staphylococcus aureus Using Specific Peptides Displayed on Yeast Vacuoles

**DOI:** 10.1128/spectrum.00920-23

**Published:** 2023-04-26

**Authors:** Jaewoong Lee, Ngoc-Tu Nguyen, Le-Minh Tran, Yang-Hoon Kim, Jiho Min

**Affiliations:** a School of Chemical Engineering, Jeonbuk National University, Deokjin-Gu Jeonju, Jeonbuk, South Korea; b Center for Ecology and Environmental Toxicology (CEET), Chungbuk National University, Seowon-Gu, Cheongju, South Korea; c School of Biological Sciences, Chungbuk National University, Seowon-Gu, Cheongju, South Korea; Connecticut Agricultural Experiment Station

**Keywords:** phage display, pathogen detection, drug carrier, *Staphylococcus aureus*, peptide conjugation, yeast vacuoles

## Abstract

Staphylococcus aureus is a common pathogen that causes health care-related and community-associated infections. In this study, we provide a novel system that can recognize and kill S. aureus bacteria. The system is specifically based on a combination of the phage display library technique and yeast vacuoles. A phage clone displaying a peptide capable of specific binding to a whole S. aureus cell was selected from a 12-mer phage peptide library. The peptide sequence was SVPLNSWSIFPR. The selected phage’s ability to bind specifically with S. aureus was confirmed using an enzyme-linked immunosorbent assay, and the chosen peptide was then synthesized. The results showed that the synthesized peptides displayed high affinity with S. aureus but low binding ability with other strains, including Gram-negative and Gram-positive bacteria such as Salmonella sp., *Shigella* spp., Escherichia coli, and Corynebacterium glutamicum. In addition, yeast vacuoles were used as a drug carrier by encapsulating daptomycin, a lipopeptide antibiotic used to treat Gram-positive bacterial infections. The expression of specific peptides at the encapsulated vacuole membrane created an efficient system that can specifically recognize and kill S. aureus bacteria.

**IMPORTANCE** The phage display method was used to select peptides with high affinity and specificity for S. aureus, and these peptides were then induced to be expressed on the surface of yeast vacuoles. These surface-modified vacuoles can act as drug carriers, with drugs such as the lipopeptide antibiotic daptomycin loaded inside. An advantage of using yeast vacuoles as a drug carrier is that they can be easily produced through yeast culture, making the approach cost-effective and suitable for large-scale production and potential implementation in clinical settings. This novel approach offers a promising way to specifically target and eliminate S. aureus that could ultimately lead to improved treatment of bacterial infections and reduced risk of antibiotic resistance.

## INTRODUCTION

Staphylococcus aureus is a Gram-positive bacterium often associated with a broad spectrum of human diseases, ranging from mild skin infections to endocarditis, sepsis, and pneumonia. Its pathogenic potential is enhanced by several bacterial virulence factors, including the presence of exotoxins and adhesins (1). It also causes infections in farm animals and contaminates primary food products, which is a risk for public health (2). The widespread habitat of S. aureus in nature makes it very difficult to control the organism, and contamination is almost impossible to prevent (3). Therefore, it is essential to develop accurate and rapid methods for detection of S. aureus to ensure human safety. Despite the recent development of detection methods without enrichment, such as real-time fluorescent quantitative PCR to detect specific nucleic acid, such methods require skilled operators and expensive equipment (4, 5). Thus, many researchers have recently geared their efforts toward developing quick, reliable strategies.

Specific biosensors have been created using antibodies, peptides, aptamers, and some proteins as targeting ligands to probe S. aureus cells (6–9). However, many of these are usually susceptible to deterioration in harsh environments, and they require laborious immobilization onto sensor substrates. Recent studies have employed phage display technology to screen new single-chain variable fragments or peptides against S. aureus from a landscape phage library. Phage display technology has been implemented in numerous fields as a valid substitute for antibodies or peptides (10–13). This technique uses a filamentous virus (commonly called bacteriophage or phage) that infects the bacterium Escherichia coli. The phage consists of a cylindrical shell mostly made of P8 (major coat protein) and P3 (minor coat protein) and encloses a circular single-stranded DNA molecule. Phage display allows the incorporation of random peptide sequences into the coat proteins. The phage pool is composed of several phages, each of which expresses only a peptide sequence (phage clones) selected against a definite target (14). Consequently, the phage can bind to a molecular target and thus can be used in therapeutic target validation, drug design, and vaccine development; it is a probe that detects specific targets in a biosensor or imaging diagnosis (14–19). Furthermore, phage-displayed peptides and phage function proteins have been successfully used as molecular recognition agents to rapidly screen numerous bacteria, including S. aureus (20).

The aim of this study was to develop a robust drug carrier to specifically recognize and kill Staphylococcus aureus, a significant pathogen that causes health care-related and community-associated infections. To achieve this goal, we selected a Ph.D.-12 phage display peptide library and used two panning methods to identify peptides that would specifically bind to the bacterium’s surface. The isolated phage clones were confirmed to interact specifically with S. aureus and demonstrated efficacy in binding properties over time using an enzyme-linked immunosorbent assay (ELISA). Upon confirming the binding affinity and specificity of the discovered peptides to S. aureus, we utilized a recombinant plasmid to express the selected peptides and conjugate them with yeast vacuoles. The resulting peptide-conjugated vacuoles were then used as a drug carrier. Daptomycin, a lipopeptide antibiotic administered to treat Gram-positive bacterial infections, including those caused by S. aureus and methicillin-resistant S. aureus (MRSA), was encapsulated into the peptide-conjugated vacuoles to create a new drug carrier for treating S. aureus infection. The vacuolar yeast membrane is composed of a single bilayer of phospholipids and cholesterol, allowing daptomycin to penetrate due to its lipophilic structure (21). The combination of daptomycin and peptide-conjugated vacuoles provides a potent drug carrier that specifically recognizes and kills S. aureus cells. Overall, the development of this novel drug carrier, which utilizes yeast vacuoles displayed with specific peptides, offers a promising approach for targeted treatment of S. aureus infections. This addresses the urgent need for more effective therapies against this pathogen, which is a significant cause of health care-related and community-associated infections.

## RESULTS AND DISCUSSION

### Selection of the S. aureus binding phage.

To identify peptides that could specifically bind to S. aureus cells, a phage display library technique was used. In this technique, a library of phages displaying random peptides on their surfaces was incubated with S. aureus cells. After several rounds of selection, phages that displayed peptides with high affinity and specificity for S. aureus cells were isolated. To determine the phage DNA sequences displayed in their major coat protein, 20 phage clones from each panning method were amplified, and their DNA was sequenced. The resulting sequences were analyzed, and different sequences were found to be present more than once, with their frequencies shown in Table 1. Finally, phages that possessed the sequences AAHRVGGFNYHM or SVPLNSWSIFPR identified by the cell fixing method or VGQFGTSQMILP identified using the sonication method were chosen for further testing of their binding affinity with S. aureus cells.

**TABLE 1:** Peptide sequences of the phage display against S. aureus

Method	Motif sequence^*a*^	Frequency	Name
Cell-fixing method	**AAHRVGGFNYHM**	7/20	SA_1
	**SVPLNSWSIFPR**	5/20	SA_2
	STVYHTTPYHNR	3/20	
Sonication method	**VGQFGTSQMILP**	6/20	SA_3
	SWPTFTVLKNHA	3/20	
	NFTLQAHPHKYP	3/20	

a Bold text indicates sequences that were chosen for further testing of their binding affinity with S. aureus cells.

### Phage binding affinity test by ELISA.

**FIG 1:**
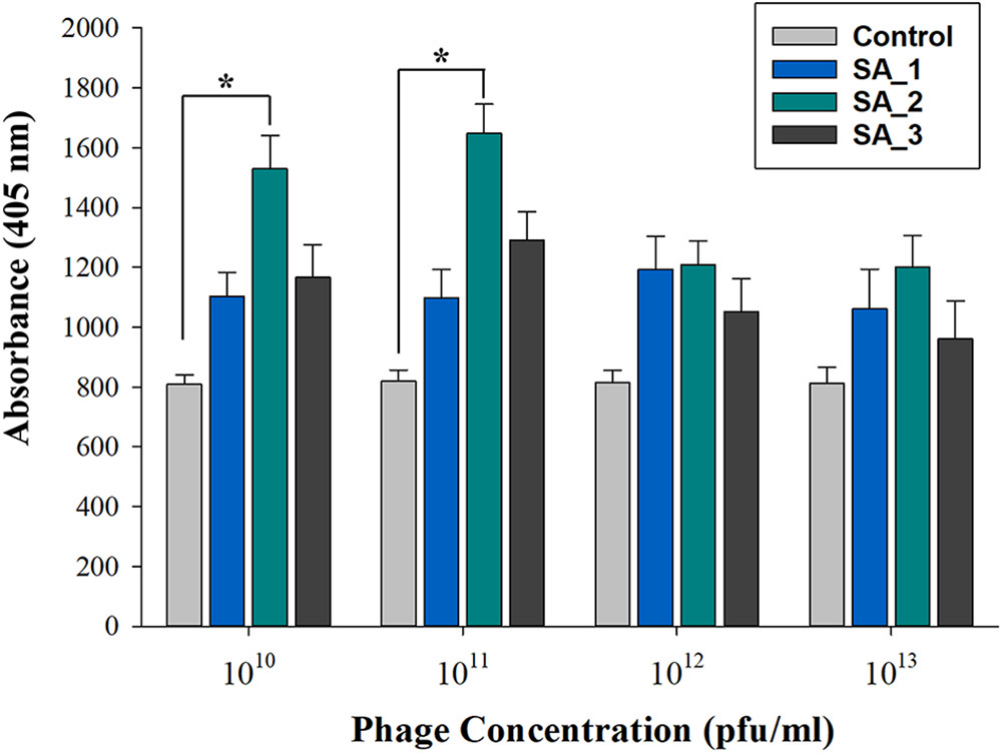
Binding affinity of selected phages to S. aureus. The data were analyzed using SigmaPlot (SPSS, Chicago, IL, USA), and a *P* value of <0.05 was considered significant. Phages SA_1 and SA_2 were isolated using the traditional panning method; phage SA_3 was isolated using the sonication method.

To analyze the specificity of the selected phages, a 96-well plate coated with an S. aureus suspension was treated with 4-fold dilutions of the phages. The bound peptides were detected using an anti-M13 antibody (anti-M13-HRP conjugate), and the signal was evaluated using the Promega fluorometer at a wavelength of 405 nm. As shown in Fig. 1, the fluorescence intensities from samples coated with the S. aureus suspension were significantly higher than those of the negative control (wells without S. aureus) for both the phages selected using the traditional panning method (SA_1, SA_2) and the phage selected from the sonication method (SA_3). SA_2 showed about 47% higher fluorescence intensity than the negative control, and SA_1 and SA_3 showed about 28% higher fluorescence intensity. The results suggest that the three selected phages were specifically bound to S. aureus and did not cross-react with the polystyrene surface. Moreover, SA_2 demonstrated a higher binding affinity at 10^10^ and 10^11^ PFU/mL than SA_1 and SA_3, while there was no significant difference at 10^12^ and 10^13^ PFU/mL (*P* > 0.05) among the three peptides. In future studies, SA_2, which exhibited the highest binding affinity to S. aureus cells, should be synthesized, and its binding affinity with other bacterial strains should be assessed.

### Specificity of synthetic peptides (SA_2) to S. aureus.

**FIG 2:**
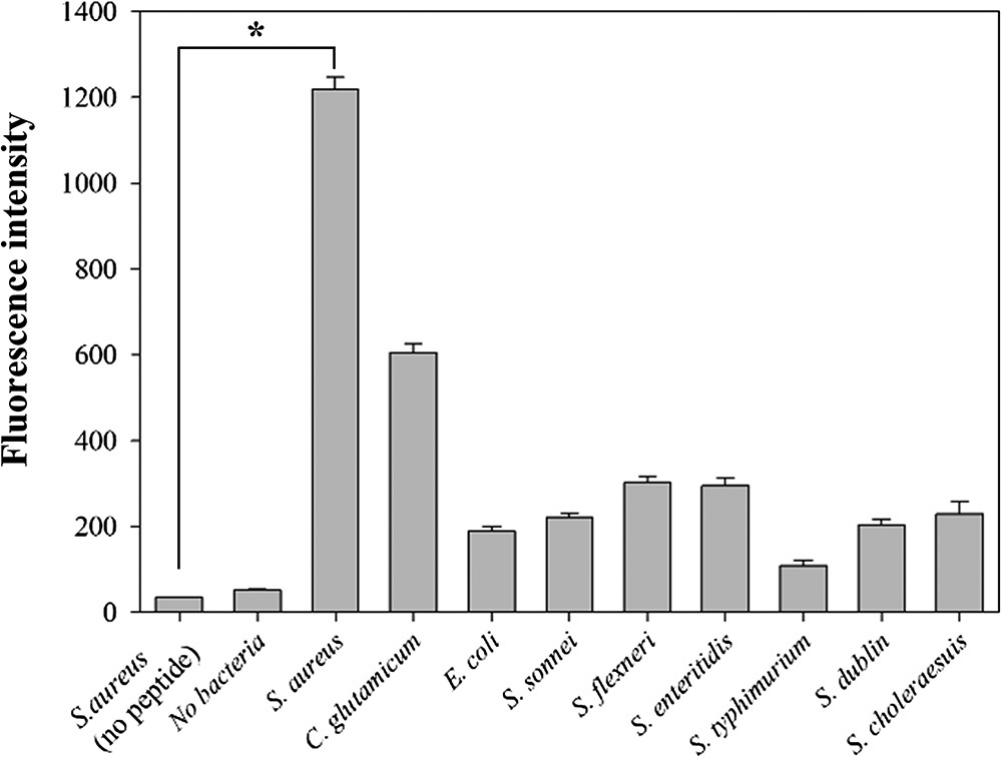
Binding affinity of SA_2 synthetic peptide to S. aureus. The data were analyzed using SigmaPlot (SPSS), and a *P* value of <0.05 was considered significant. (Gram positive, S. aureus and C. glutamicum; Gram negative, Salmonella spp. and *Shigella* spp.)

To validate the specificity of SA_2, the peptide was labeled with carboxyfluorescein (FAM) and tested against various bacterial strains, including Gram-negative and Gram-positive species. As shown in Fig. 2, the binding affinity of SA_2 to S. aureus cells was about 50% higher than that to C. glutamicum cells, which was the second highest of the tested strains, and about 80% higher than other control groups on average. In contrast, the fluorescence intensities were much lower for Gram-negative bacteria, and even these signals were still higher than those of the control. These findings provide evidence that SA_2 is highly specific for Gram-positive bacteria, especially S. aureus. Moreover, among the Gram-positive strains, SA_2 displayed the highest binding affinity to S. aureus cells, with a 2-fold higher fluorescence intensity than that for C. glutamicum. This indicates that SA_2 can selectively recognize and bind to S. aureus cells with high specificity, making it a promising candidate for the development of new treatments against S. aureus infections.

### Antimicrobial activity of SA_2 peptide.

**FIG 3:**
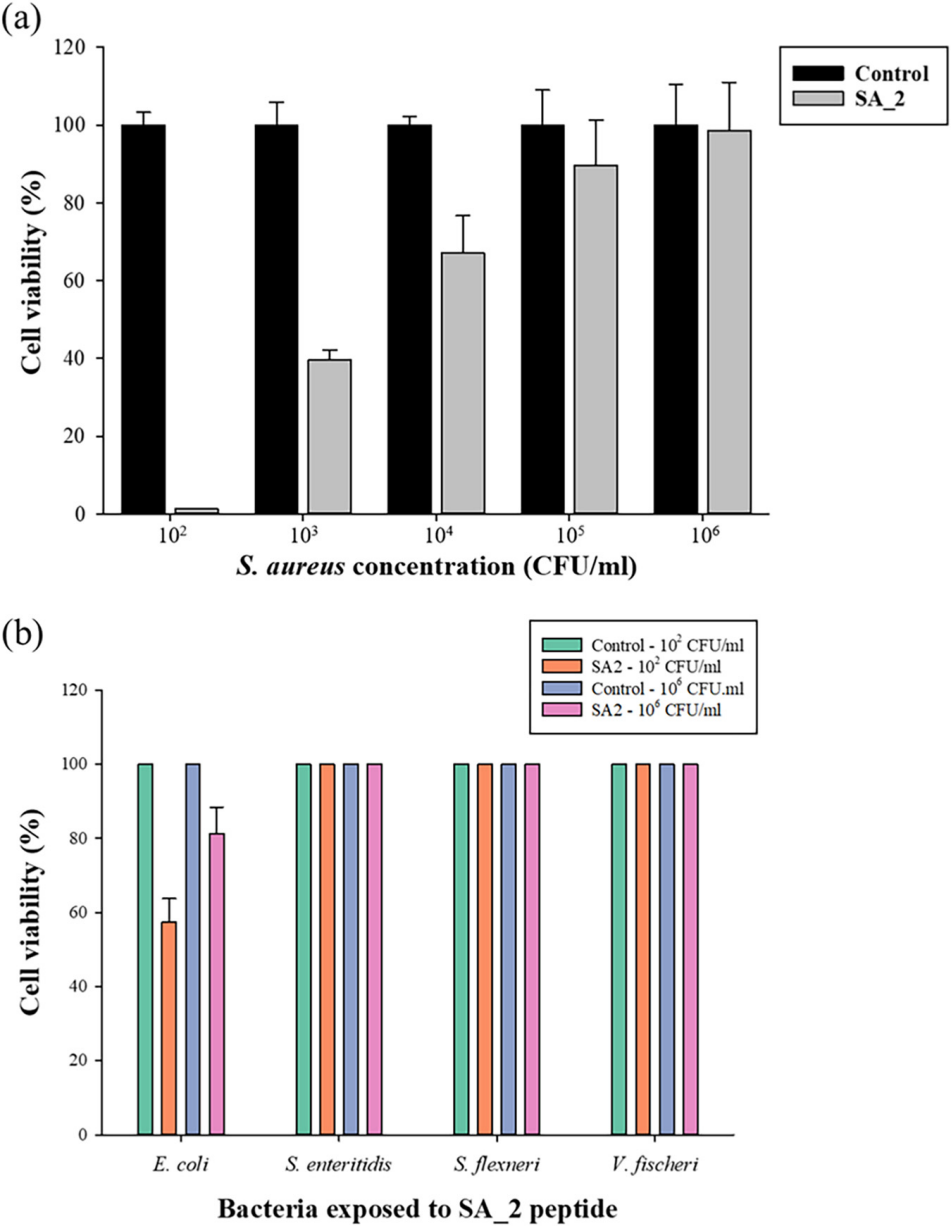
The effect of SA_2 peptide on the growth of S. aureus (a) and E. coli, S. enteritidis, S. flexneri, and V. fischeri (b) at 10^2^ CFU/mL and 10^6^ CFU/mL. SA_2 peptide does not show a significant antimicrobial effect against Gram-negative bacteria, and the effect is more pronounced at low CFU.

To confirm the antibacterial activity of the SA_2 peptide, which specifically binds to S. aureus, we analyzed the degree of cell growth inhibition against S. aureus and several other bacteria. Initially, we exposed different concentrations of SA_2 (20 μg/mL) to various concentrations of S. aureus (10^2^ to 10^6^ CFU/mL) to determine its impact on bacterial growth. As shown in Fig. 3a, at 10^2^ CFU/mL, SA_2 killed 98.7% of the bacteria, while at higher concentrations, the bactericidal effect decreased significantly. Additionally, we exposed SA_2 to different bacteria, including E. coli, Salmonella enterica serovar Enteritidis, Shigella flexneri, and Vibrio fischeri, at 10^2^ and 10^6^ CFU/mL. The results depicted in Fig. 3b showed that SA_2 had a slight bactericidal effect on E. coli at 10^2^ CFU/mL (47% of bacteria were killed) and 10^6^ CFU/mL (19% of bacteria were killed), while it did not affect the growth of the other bacteria. These findings indicate that SA_2 possesses a high level of specificity and selective antimicrobial activity against S. aureus, making it a promising candidate for the development of new treatments for S. aureus infections.

### SA_2 peptides displayed on yeast vacuoles.

To simplify the synthesis of the SA_2 peptide, which has specific binding and antibacterial activity against S. aureus, we proposed the use of yeast vacuoles. These vacuoles contain various types of hydrolytic enzymes and are used as antibacterial or antiviral agents. If these vacuoles and the SA_2 peptide are combined and used as a single material, it may be possible to create an antibacterial agent that specifically targets S. aureus. This approach could potentially lead to the development of new treatments for S. aureus infections and may also help reduce the risk of antibiotic resistance.

**FIG 4:**
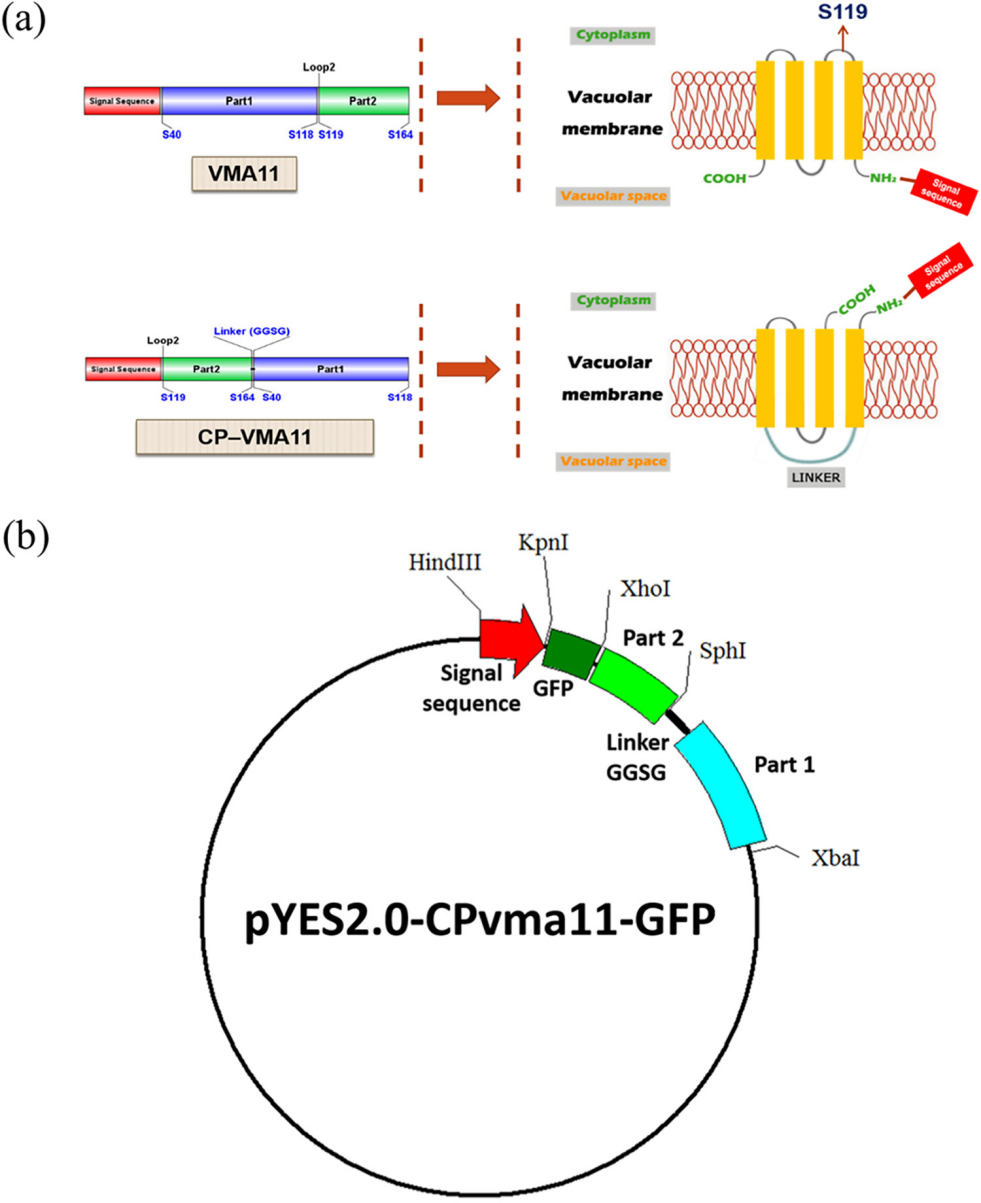
(a) Mechanism of the permutation between parts 1 and 2, along with the addition of linker (GGSG) between the N and C termini of original VMA11 can create newborn N and C termini, both exposed to the cytoplasm; (b) plasmid pYES2-CPvma11-GFP that contains a signal sequence which leads the peptide of interest to the vacuolar membrane.

To express the SA_2 peptide on the yeast vacuolar membrane, a specific plasmid was constructed from the original plasmid pYES2 (22). As described in Fig. 4a and b, the plasmid pYES2-CPvma11-GFP contains a signal sequence (SS) fragment, part 1 (P1) fragment, and part 2 (P2) fragment, which were prepared via PCR with the oligonucleotide primers described in Table 2. VMA11 is a vacuolar membrane ATPase (V-ATPase) in Saccharomyces cerevisiae that consists of two separable domains: peripheral catalytic domains (V1) and integral membrane domains (V0). This ATPase complex belongs to a vacuolar-type proton pump that functions in vacuole acidification and energy generation on the membrane (23). Among different V-ATPase components, VMA11 has been shown to be required for the operation and assembly of the V-ATPase complex. More specifically, the Vma11p gene encodes the VMA11 polypeptide or c′ subunit of the yeast V-ATPase V0 domain. In this plasmid, the fragments SS, P1, and P2 are different parts of the polypeptide VMA11. Therefore, the peptide of interest, cloned behind the signal sequence and transformed into the yeast, will be expressed on the vacuolar membrane. In this work, we inserted the DNA sequence of the SA_2 peptide (AGCGTGCCCCTGAACAGCTGGAGCATCTTCCCCAGG) between SS and GFP and then transformed the recombinant plasmid into S. cerevisiae (ATCC 208280). After that, the vacuole that contained SA_2 on the membrane was isolated, and the antimicrobial activity was confirmed. Figure S1 in the supplemental materials shows a fluorescence picture of S. cerevisiae cells genetically engineered to express the SA_2 peptide on the surface of vacuoles.

**TABLE 2:** All oligonucleotide primers used in this study

Oligonucleotide primer^*a*^	Sequence^*b*^	Source and purpose
SS-F	GGCAAGCTTATGTCAACGCAACTCGCA	F primer for pYES2-SS
SS-R	ATAGGTACCAGCTGTACCAATGGCAGC	R primer for pYES2-SS
P2-F	ATACTCGAGGTCGGTGACGTTGGTGTT	F primer for pYES2-SS-P2
P2-R	GTTGCATGCTTCAGAGCCTCTAGTGTT	R primer for pYES2-SS-P2
P1-F	GTAGCATGCCCACCAAGACCAAAGTCAGGTATTG	F primer for pYES2.0-CPvma11
P1-R	GAGTCTAGATCACATACCAATGGCGTAGCC	R primer for pYES2.0-CPvma11
c4-GFP-F	TATGGTACCGTGAGCAAGGGCGAGGAG	F primer for pYES2.0-CPvma11-GFP
c4-GFP-R	GTACTCGAGCTTGTACAGCTCGTCCAT	R primer for pYES2.0-CPvma11-GFP
SA-F	CAGCGTGCCCCTGAACAGCTGGAGCATCTTCCCCAGGGGTAC	F primer for oligonucleotide annealing of SA_2
SA-R	CCCTGGGGAAGATGCTCCAGCTGTTCAGGGGCACGCTGGTAC	R primer for oligonucleotide annealing of SA_2

a F, forward; R, reverse.

b Underlines indicate restriction enzyme sites.

**FIG 5:**
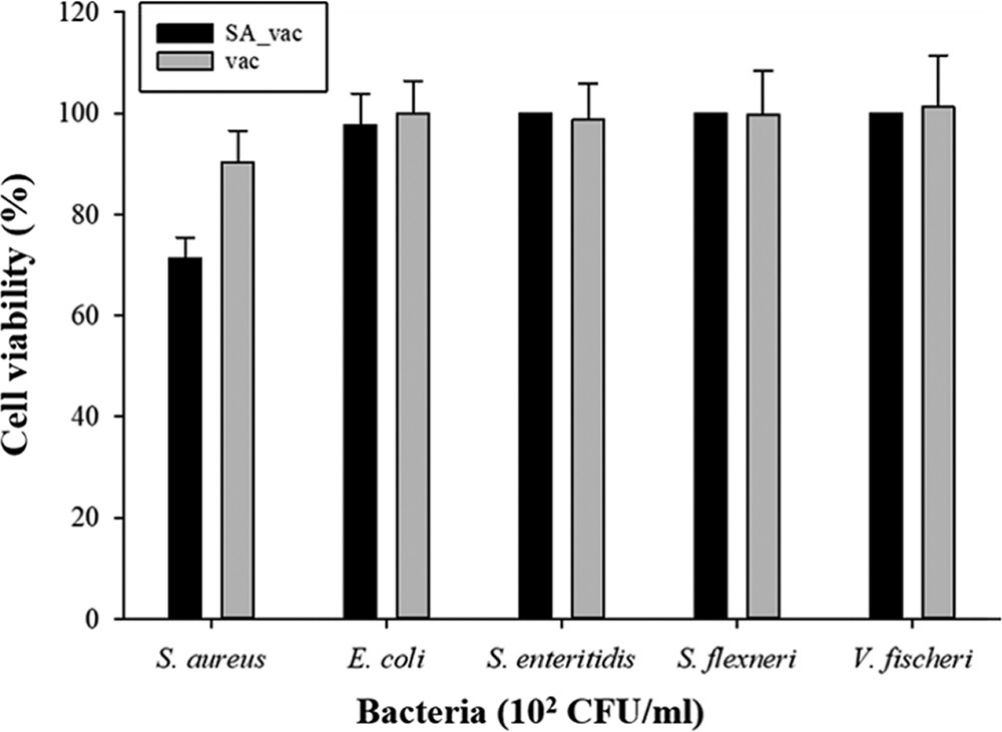
Antimicrobial effect of wild-type yeast vacuoles (vac) and peptide-conjugated vacuoles (SA_vac) on various bacterial strains (S. aureus, E. coli, S. enteritidis, S. flexneri, V. fischeri).

In this experiment, various concentrations of vacuoles (5%, 10%, 20%, 30% [wt/vol]) were exposed to S. aureus at 10^6^ CFU/mL to determine the optimal concentration of the vacuole from recombinant yeast. The yeast vacuoles did not show a high antimicrobial effect (data not shown). Therefore, we compared the antibacterial activity of wild-type vacuoles and recombinant vacuoles designed to express SA_2 on the surface and that have specific binding ability to S. aureus. Upon exposure to a high concentration of S. aureus (10^6^ CFU/mL), there was no difference observed between the wild-type yeast vacuoles (referred to as “vac”) and the recombinant yeast vacuoles (referred to as “SA_vac”) (data not shown). Hence, to confirm the activities of SA_vac at a lower concentration of S. aureus (10^2^ CFU/mL) and other bacteria, we conducted further analysis. The results (depicted in Fig. 5) indicated no antimicrobial effect of the vacuoles on E. coli, S. Enteritidis, S. flexneri, and V. fischeri. As for S. aureus, the antimicrobial activity of SA_vac decreased slightly when treated with a lower concentration of S. aureus (35% bacteria-killing effect at 10^2^ CFU/mL of S. aureus). This decrease could be attributed to the fact that a lower density of bacteria could impede the vacuole’s ability to bind to the bacteria. Interestingly, when exposed to S. aureus, the wild-type yeast vacuole activity was lower than that of SA_vac, which emphasized the role of the SA_2 peptide. The higher effect of the SA_vac demonstrated that vacuoles with SA_2 could bind and kill bacteria more accurately than typical vacuoles. Therefore, it was confirmed that while vac showed no antibacterial activity against all the bacteria tested in this experiment, the SA_2 peptide expressed on the surface of SA_vac had both specificity and antibacterial activity against S. aureus.

### Antimicrobial activity of daptomycin-encapsulated vacuoles.

**FIG 6:**
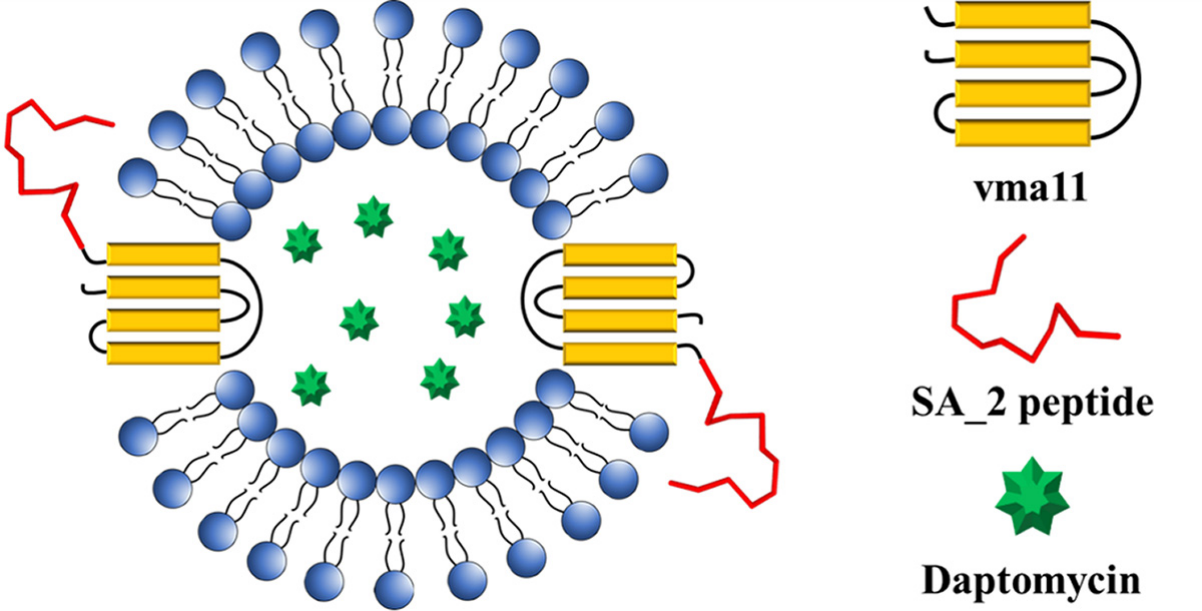
Scheme of daptomycin-encapsulated vacuoles (SA_vac^Dap^).

**FIG 7:**
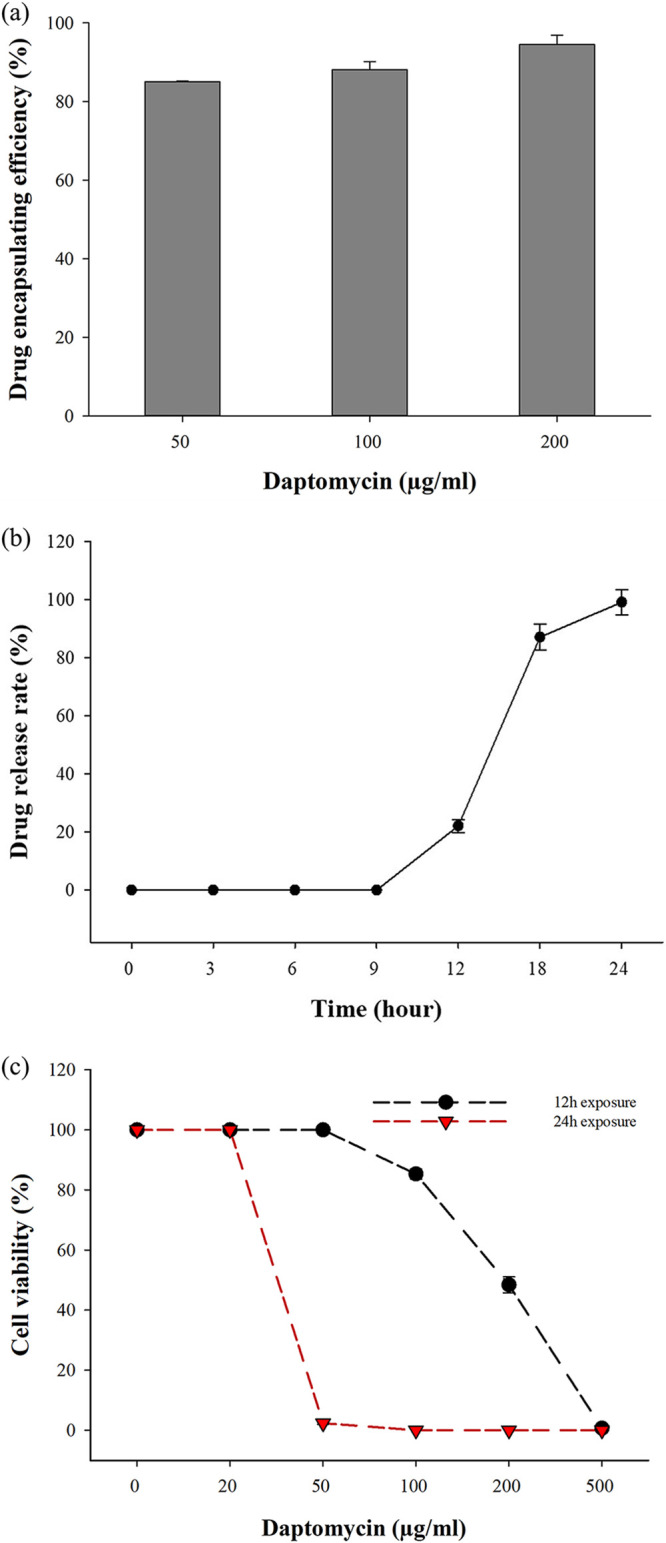
(a) Drug encapsulating efficiency of daptomycin in SA_vac. (b) Drug release rate of daptomycin-encapsulated vacuoles (SA_vac^Dap^). (c) Antimicrobial activity of daptomycin-encapsulated vacuoles.

Daptomycin is a lipopeptide antibiotic commonly used to treat Gram-positive bacterial infections, especially S. aureus and methicillin-resistant S. aureus (MRSA) (23). In this study, we attempted to encapsulate daptomycin into SA_vac to create a new drug carrier for S. aureus treatment. The yeast vacuolar membrane is composed of a single bilayer made of phospholipids and cholesterol, allowing daptomycin penetration due to its lipophilic structure (24). First, we constructed a standard curve by evaluating the absorbance of standard daptomycin at several concentrations. Daptomycin was dissolved in ethanol, and its absorbance was measured at 220.5 nm using a spectrophotometer (Fig. S2a). Next, we evaluated the impact of daptomycin on the growth of S. aureus cells. At 10 μg/mL of daptomycin, 99% of the S. aureus colony was killed, while at 20 μg/mL of daptomycin, no living bacteria could be observed (Fig. S2b). Therefore, we defined 20 μg/mL as the MIC of daptomycin for S. aureus in this study. Next, we used SA_vac as a drug carrier for daptomycin. Since SA_vac contains the specific peptide for S. aureus, the conjugation of daptomycin, SA peptide, and vacuoles could be a potent agent for S. aureus and MRSA treatment (Fig. 6). Various concentrations of daptomycin (50, 100, 200 μg/mL) were mixed with 30% vacuoles, and the final volume was adjusted to 1 mL using PS buffer (10 mM PIPES KOH [piperazine-*N*,*N*′-bis(2-ethanesulfonic acid) KOH] [pH 6.8], 200 mM sorbitol). The results, shown in Fig. 7a, demonstrated that daptomycin was entirely loaded into the vacuoles, with 50, 100, and 200 μg/mL of daptomycin having loading efficiencies of 85%, 88%, and 94.5%, respectively. The amount of drug loaded in the vacuoles increased with increasing initial daptomycin concentration, suggesting a concentration gradient-mediated drug loading. We then analyzed the drug efflux to confirm the release kinetic. The vacuoles that encapsulated 200 μg/mL of daptomycin were transferred to a dialysis tube, and the release rate was analyzed. As shown in Fig. 7b, the drug started to release after 12 h of shaking, with a release rate of 22.11%. After 18 h and 24 h, the leak rate increased to 87.16% and 99.19%, respectively. These results indicate that the vacuole structure becomes destabilized or disrupted after 12 h of shaking in phosphate-buffered saline (PBS) (pH 7.4). In addition, the delayed release of daptomycin out of the vacuole gives the developed vacuoles time to reach the target sufficiently, and it is thought that it can provide a little more target-specific antibacterial activity.

In this study, it was demonstrated that the encapsulation of daptomycin into SA_vac could potentially create a new drug carrier for the treatment of S. aureus and MRSA infections. The drug was efficiently loaded into vacuoles, and the amount of drug loaded increased with an increasing initial daptomycin concentration, indicating a concentration gradient-mediated drug-loading mechanism. The release kinetic study showed that daptomycin was gradually released from the vacuoles in a time-dependent manner, and the release rate increased with increasing daptomycin concentration. When the daptomycin-encapsulated vacuoles (SA_vac^Dap^) were exposed to S. aureus, the results showed that their antimicrobial activity was dependent on the drug concentration and exposure time. The 24-h exposure experiment demonstrated that the daptomycin-encapsulated vacuoles had a higher antimicrobial activity against S. aureus, killing 98% of the bacteria with 50 μg/mL of loaded daptomycin due to a 100% release rate after 24 h. These findings suggest that SA_vac^Dap^ could be a potent agent for the treatment of S. aureus and MRSA infections, especially when used in combination with other therapeutic agents.

This study provides a new strategy for the development of a drug delivery system that could potentially enhance the therapeutic efficacy of antibiotics against S. aureus and MRSA infections. Further studies are needed to optimize the loading efficiency, drug release rate, and therapeutic efficacy of SA_vac^Dap^ and to evaluate its safety and pharmacokinetics *in vivo*.

### Conclusion.

This study presents the first report of a drug carrier using yeast vacuoles conjugated with a phage peptide technique to create a specific detecting and eliminating agent for S. aureus. The discovered peptide SA_2 exhibited high specificity to S. aureus and was successfully conjugated with the vacuolar yeast membrane to form a drug carrier named SA_vac. SA_vac encapsulated daptomycin, a lipopeptide antibiotic widely used to treat both S. aureus and MRSA infections, due to the vacuolar membrane characteristics. The study achieved a perfect encapsulation efficiency of daptomycin by SA_vac, with a maximum of 94.5%, and maintained the drug for 12 h before releasing it outside. The daptomycin-encapsulated vacuoles (SA_vac^Dap^) exhibited high bactericidal activity that killed most of the S. aureus cells after 12 h of treatment at a dose of 500 μg/mL, or after 24 h at a dose of 50 to 200 μg/mL. Although we were unable to proceed with *in vivo* verification due to a lack of environment and equipment, and the safety and stability of vacuoles bound to specific peptides could not be observed, the positive trend in the results suggests that this approach could become a more effective drug delivery system. Furthermore, since daptomycin can also be used for the treatment of MRSA, the strategy used in this study is expected to be applied to MRSA infections and other drug delivery applications. Further studies should be conducted to verify the stability and safety of the vacuoles and their bound peptides *in vivo* and to improve the efficiency of this promising approach.

## MATERIALS AND METHODS

### Phage library and strains.

The Ph.D. 12-mer phage library was purchased from New England Biolabs (Beverly, MA, USA), while the anti-M13 antibody (B62-FE2) (horseradish peroxidase [HRP]) was purchased from Abcam (Cambridge, MA, USA). The phage display system is based on an M13 phage vector modified to display the peptide as N-terminal fusions to the coat protein pIII, with a short linker sequence (Gly-Gly-Gly-Ser) between the displayed peptide and pIII. Escherichia coli ER2738 host strains were provided by the Ph.D. peptide display cloning system. Staphylococcus aureus (ATCC 6538) and Saccharomyces cerevisiae 2805 (ATCC 208280) were provided by the Korean Research Institute of Bioscience and Biotechnology (KRIBB; Daejeon, South Korea) and grown in YPD medium (5 g/L yeast extract, 10 g/L tryptone, 10 g/L sodium chloride) in a 250-mL flask at 37°C and 180 rpm (25, 26). Other bacterial strains, including Salmonella enterica serovar Choleraesuis ATCC 10708, S. Enteritidis ATCC 13076, S. enterica serovar Typhimurium KTCC 2053, S. enterica serovar Dublin ATCC 15480, Shigella sonnei ATCC 25931, Shigella flexneri KCTC 2517, E. coli BL21(DE3), Corynebacterium glutamicum ATCC 13032, and Vibrio fischeri ATCC 49387, were provided by the Department of Microbiology at Chungbuk National University (South Korea) and grown in LB medium (10 g/L yeast extract, 20 g/L peptone, 20 g/L glucose) in a 250-mL flask at 30°C and 180 rpm.

### Two panning methods for phage selection.

**(i) Cell-fixing method.** Staphylococcus aureus cells were cultured in Luria-Bertani (LB) medium until the optical density at 600 nm (OD_600_) reached 0.6 to 0.8. The cells were then collected by centrifugation, washed with phosphate-buffered saline (PBS), and mixed with ethanol before being dried on a 96-well plate (100 μL/well) at room temperature (RT) overnight. For the first panning round, the cells fixed on the 96-well plate were exposed to 10^11^ PFU/mL of phage library in TBST buffer (Tris-buffered saline with 0.05% Tween 20, pH 7.5) (100 μL/well). Unbound phages were eliminated by washing five times with TBST, and the bound phages were eluted with 100 μL elution buffer (0.2 M Glycine-HCl) for 20 min at room temperature. The eluted phage was neutralized with 1 M Tris-HCl (pH 9.1) and amplified in E. coli ER2738, followed by purification with polyethylene glycol precipitation (26).

**(ii) Sonication method.** The 10^11^ PFU/mL of Ph.D.-12 phages was exposed to S. aureus cells (OD_600_, 0.5) in PBS for 60 min at RT with gentle agitation. The bacteria-phage mixture was centrifuged for 5 min at 16,000 × *g*, and then the unbound phages were removed by washing 10 times (centrifugation at 16,000 × *g* for 5 min with 1 mL TBST). The pellet that contained the bound phages was eluted with 250 μL of 0.2 M glycine-HCl (pH 2.2) with slight shaking at RT for 20 min and was then sonicated for 10 min with 50% amplitude, 30 s on and 30 s off. Subsequently, the solutions were neutralized with 25 μL of 1 M Tris-HCl (pH 9) (26). The eluate (10 μL) would be used for phage titering, and 500 μL was used to infect E. coli ER2738 for amplification (26).

### Phage titration and amplification.

Serial dilutions of the phage were prepared as follows: 10^8^ to 10^11^ for amplified phage culture supernatants and 10^1^ to 10^4^ for the unamplified panning eluates. E. coli ER2738 was cultured in LB medium until the OD_600_ value increased to 0.5, and then 200 μL of bacterial culture was mixed with 10 μL of each phage dilution at RT for 5 min before transferral to top agar (10 g/L tryptone, 5 g/L yeast extract, 5 g/L NaCl, 7 g/L agar). After that, the top agar was instantly poured onto an LB/IPTG/X-gal plate and incubated at 37°C overnight (o/n), and the PFU was subsequently determined through the blue plaques quantity.

For phage amplification, ER2738 was cultured with tetracycline o/n and then diluted in 20 mL LB medium (1:100) before adding unamplified eluate phage and incubating the culture with shaking at 180 rpm and 37°C for 4.5 h. After that, the culture was centrifuged for 10 min at 12,000 × *g* and 4°C, and 80% of the supernatant was transferred to a fresh tube with 20% polyethylene glycol (PEG) and 2.5 M NaCl. The phage was precipitated o/n at 4°C and was then spun at 12,000 × *g* and 4°C for 15 min. Subsequently, the supernatant was transferred to a fresh microcentrifuge tube and was reprecipitated by adding 1/6 volume of 20% PEG/2.5 M NaCl before incubation on ice for 60 min. The sample was then centrifuged at 14,000 rpm and 4°C for 10 min, followed by removal of the supernatant. Finally, the pellet was suspended in 200 μL TBS and was centrifuged for 1 min to pellet any remaining insoluble material. Then, the supernatant that contained amplified eluate was transferred to a fresh tube. For ELISA or sequencing of the plaque, strain ER2738 was cultured and then diluted in LB medium (1:100) before mixing with the blue plaque collected from the plate. The tube was incubated at 37°C with shaking for 4.5 to 5 h and then placed in the microcentrifuge at 14,000 rpm for 30 s. After that, 80% of the supernatant was transferred to a fresh tube as the amplified phage stock (25, 26).

### Sequencing of the phage DNA.

The phage-containing supernatant (500 μL) was transferred to a fresh tube before adding 200 μL of 20% PEG/2.5 M NaCl. The tube was inverted several times, was let stand for 20 min at RT, and then placed in a microcentrifuge at 14,000 rpm and 4°C for 10 min. The supernatant was discarded while the pellet was suspended thoroughly in 100 μL of iodide buffer. Afterward, 250 μL of ethanol was added, and the tube was spun at 14,000 rpm for 10 min at 4°C. The pellet was washed with 0.5 mL of 70% ethanol after discarding the supernatant, finally suspended in 30 μL distilled water (DW), and stored at −20°C. In addition, all sequences were analyzed by Bioneer (Daejeon, South Korea).

### Phage binding affinity test.

An ELISA plate was coated with an S. aureus suspension using the methods described in “Two panning methods for phage selection,” above. Serial dilutions of the phage in 100 μL of TBST were prepared, added to each well, and then incubated at RT for 1 h with agitation. The plate was washed six times with TBST; then, 200 μL of the diluted HRP-conjugated anti-M13 monoclonal antibody (GE Healthcare) was added to each well and incubated at RT for 1 h with agitation. The HRP substrate solution was prepared according to the manufacturer’s instructions. Finally, 200 μL of substrate solution was added to each well and incubated for 60 min at room temperature (RT) with gentle agitation, and the plate was read at 405 to 415 nm.

### Synthetic peptide affinity test.

The peptide SA_2 was synthesized and labeled with carboxyfluorescein (FAM) for observing the peptide binding. In the synthetic peptide affinity test, a black 96-well plate was coated with various bacteria using the cell-fixing method described above. S. Choleraesuis ATCC 10708, S. Enteritidis ATCC 13076, *S.* Typhimurium KTCC 2053, *S.* Dublin ATCC 15480, S. sonnei ATCC 25931, S. flexneri KCTC 2517, E. coli BL21(DE3), and C. glutamicum ATCC 13032 were used in the synthetic peptide affinity test. The peptide SA_2 was exposed to different bacteria, including Gram-negative and Gram-positive strains, to confirm its specificity and then incubated at RT for 1 h with agitation. The fluorescence intensity from carboxyfluorescein (FAM) in the peptide SA_2 was then evaluated (excitation, 475 nm; emission, 500 to 550 nm) using the GloMax Explorer microplate reader (Promega).

### Vacuole isolation from S. cerevisiae.

The isolation method was as described above. The yeast was cultured and harvested at the exponential phase. Then, 5 mL of the cell suspension was added to 25 mL of 100 mM Tris-SO_4_ buffer, pH 9.4 (containing 10 mM dithiothreitol [DTT]), followed by incubation for 15 min at 30°C. After that, the mixture was centrifuged at 1,610 × *g* and 4°C for 5 min. The supernatant was removed, and the pellet was collected and suspended in 5 mL of breaking buffer (containing 20 mM Tris-Cl [pH 7.4] and 0.6 M sorbitol). In the next step, the cells were lysed by adding 1 g of glass beads to the cell mixture before vortexing it five times, for 30 s each. The lysate was centrifuged at 500 × *g* for 5 min, and the supernatant was then centrifuged at 20,000 × *g* and 4°C for 30 min. The vacuoles (pellet) were finally stored at −20°C (27).

### Antimicrobial test.

An antimicrobial test was conducted to assess the antimicrobial activity of the vacuoles and synthetic peptides (28). Various bacteria were cultured until the OD_600_ reached 0.6 to 0.7, and the vacuoles (0% to 30% [wt/vol]) and synthetic peptides (0 to 50 μg/mL) were diluted in sterile water. Then, 100 μL of bacterial culture was simultaneously spread with the vacuole or peptide onto an LB plate. The antimicrobial activity was measured using the colony counts. The results are expressed as the cell viability percentage.

### Drug-encapsulating efficiency.

In this study, daptomycin was purchased from Selleck Chemicals (Seoul, South Korea). Various concentrations of daptomycin (50, 100, 200 μg/mL) were mixed with 30% vacuoles, and the final volume was adjusted to 1 mL using PS buffer (10 mM PIPES KOH [pH 6.8], 200 mM sorbitol). Encapsulation was achieved via slow mixing overnight at 4°C. The unloaded drug was removed via centrifugation at 20,000 × *g* for 30 min, and the pellet was then washed five times with PS buffer and concentrated to 1 mL. The drug-encapsulating efficiency (DEE) was calculated using the following equation (29):

$$\text{DEE }(\text{\%})\;=\;\frac{\text{Input antibiotic}\;-\;\text{Remaining antibiotic}}{\text{Input antibiotic}}\;\times \;100$$

The daptomycin concentration was read on a UV-visible (UV-vis) spectrophotometer (Mecasys, Daejeon, South Korea) by absorbance that was measured at 220.5 nm (28).

### Drug release kinetics.

Vacuoles encapsulating 200 μg/mL of daptomycin were transferred to a dialysis tube (Float-A-Lyzer, Spectrum Laboratories, Inc.). The molecular mass cutoff was set to 100 kDa, and the vacuoles were immersed in 40 mL of PBS (pH 7.4) at 37°C with gentle shaking (90 rpm). Buffer was collected for absorbance analysis every 3 h, and the amount of daptomycin released was determined by the spectrophotometer at 220.5 nm (30).

### Data availability.

The data supporting the findings of this study are available within the article and its supplemental material. Data not shown are available upon reasonable request.

## References

[1] Balasubramanian D, Harper L, Shopsin B, Torres VJ. 2017. Staphylococcus aureus pathogenesis in diverse host environments. Pathog Dis 75:ftx005. doi:10.1093/femspd/ftx005. 28104617 PMC5353994

[2] Schirone M, Visciano P, Tofalo R, Suzzi G. 2019. Foodborne pathogens: hygiene and safety. Front Microbiol 10:1974. doi:10.3389/fmicb.2019.01974. 31507576 PMC6718918

[3] Tong SY, Davis JS, Eichenberger E, Holland TL, Fowler VG. 2015. Staphylococcus aureus infections: epidemiology, pathophysiology, clinical manifestations, and management. Clin Microbiol Rev 28:603–661. doi:10.1128/CMR.00134-14. 26016486 PMC4451395

[4] Sartori C, Boss R, Ivanovic I, Graber H. 2017. Development of a new real-time quantitative PCR assay for the detection of Staphylococcus aureus genotype B in cow milk, targeting the new gene adlb. J Dairy Sci 100:7834–7845. doi:10.3168/jds.2017-12820. 28755929

[5] Galia L, Ligozzi M, Bertoncelli A, Mazzariol A. 2019. Real-time PCR assay for detection of Staphylococcus aureus, Panton-Valentine leucocidin and methicillin resistance directly from clinical samples. AIMS Microbiol 5:138–146. doi:10.3934/microbiol.2019.2.138. 31384708 PMC6642910

[6] Alamer S, Chinnappan R, Zourob M. 2017. Development of rapid immuno-based nanosensors for the detection of pathogenic bacteria in poultry processing plants. Procedia Technol 27:23–26. doi:10.1016/j.protcy.2017.04.012.

[7] Li J-N, Wang H, Han Y-X, Zhao Y-T, Zhou H-H, Xu J, Li L. 2019. Novel peptides screened by phage display peptide library can mimic epitopes of the FnBPA-A protein and induce protective immunity against Staphylococcus aureus in mice. Microbiologyopen 8:e910. doi:10.1002/mbo3.910. 31452334 PMC6813446

[8] Zhang Y, Lai BS, Juhas M. 2019. Recent advances in aptamer discovery and applications. Molecules 24:941. doi:10.3390/molecules24050941. 30866536 PMC6429292

[9] Yue H, Zhou Y, Wang P, Wang X, Wang Z, Wang L, Fu Z. 2016. A facile label-free electrochemiluminescent biosensor for specific detection of Staphylococcus aureus utilizing the binding between immunoglobulin G and protein A. Talanta 153:401–406. doi:10.1016/j.talanta.2016.03.043. 27130134

[10] Liu P, Han L, Wang F, Petrenko VA, Liu A. 2016. Gold nanoprobe functionalized with specific fusion protein selection from phage display and its application in rapid, selective and sensitive colorimetric biosensing of Staphylococcus aureus. Biosens Bioelectron 82:195–203. doi:10.1016/j.bios.2016.03.075. 27085951

[11] Petrenko VA. 2018. Landscape phage: evolution from phage display to nanobiotechnology. Viruses 10:311. doi:10.3390/v10060311. 29880747 PMC6024655

[12] Pini A, Giuliani A, Falciani C, Runci Y, Ricci C, Lelli B, Malossi M, Neri P, Rossolini GM, Bracci L. 2005. Antimicrobial activity of novel dendrimeric peptides obtained by phage display selection and rational modification. Antimicrob Agents Chemother 49:2665–2672. doi:10.1128/AAC.49.7.2665-2672.2005. 15980334 PMC1168694

[13] Sainath Rao S, Mohan KV, Atreya CD. 2013. A peptide derived from phage display library exhibits antibacterial activity against E. coli and Pseudomonas aeruginosa. PLoS One 8:e56081. doi:10.1371/journal.pone.0056081. 23409125 PMC3569419

[14] Peltomaa R, Benito-Peña E, Barderas R, Moreno-Bondi MC. 2019. Phage display in the quest for new selective recognition elements for biosensors. ACS Omega 4:11569–11580. doi:10.1021/acsomega.9b01206. 31460264 PMC6682082

[15] Saw PE, Song E-W. 2019. Phage display screening of therapeutic peptide for cancer targeting and therapy. Protein Cell 10:787–807. doi:10.1007/s13238-019-0639-7. 31140150 PMC6834755

[16] Takakusagi Y, Takakusagi K, Sakaguchi K, Sugawara F. 2020. Phage display technology for target determination of small-molecule therapeutics: an update. Expert Opin Drug Discov 15:1199–1211. doi:10.1080/17460441.2020.1790523. 32660284

[17] Mimmi S, Maisano D, Quinto I, Iaccino E. 2019. Phage display: an overview in context to drug discovery. Trends Pharmacol Sci 40:87–91. doi:10.1016/j.tips.2018.12.005. 30606501

[18] Aghebati-Maleki L, Bakhshinejad B, Baradaran B, Motallebnezhad M, Aghebati-Maleki A, Nickho H, Yousefi M, Majidi J. 2016. Phage display as a promising approach for vaccine development. J Biomed Sci 23:66. doi:10.1186/s12929-016-0285-9. 27680328 PMC5041315

[19] Hess KL, Jewell CM. 2020. Phage display as a tool for vaccine and immunotherapy development. Bioeng Transl Med 5:e10142. doi:10.1002/btm2.10142. 31989033 PMC6971447

[20] Li C, Li J, Xu Y, Zhan Y, Li Y, Song T, Zheng J, Yang H. 2021. Application of phage-displayed peptides in tumor imaging diagnosis and targeting therapy. Int J Pept Res Ther 27:587–595. doi:10.1007/s10989-020-10108-5. 32901205 PMC7471523

[21] Gujrati V, Lee M, Ko Y-J, Lee S, Kim D, Kim H, Kang S, Lee S, Kim J, Jeon H, Kim SC, Jun Y, Jon S. 2016. Bioengineered yeast-derived vacuoles with enhanced tissue-penetrating ability for targeted cancer therapy. Proc Natl Acad Sci USA 113:710–715. doi:10.1073/pnas.1509371113. 26715758 PMC4725537

[22] Tótoli EG, Salgado HRN. 2015. A green approach for the quantification of daptomycin in pharmaceutical formulation by UV spectrophotometry. Braz J Pharm Sci 51:811–821. doi:10.1590/S1984-82502015000400007.

[23] Nguyen N-T, Park R-M, Kim Y-H, Min J. 2018. Detection and discrimination of Shigella sonnei and Shigella flexneri based on vacuolar responses in Saccharomyces cerevisiae. J Biotechnol 287:1–7. doi:10.1016/j.jbiotec.2018.09.009. 30261194

[24] Hirata R, Graham LA, Takatsuki A, Stevens TH, Anraku Y. 1997. VMA11 and VMA16 encode second and third proteolipid subunits of the Saccharomyces cerevisiae vacuolar membrane H^+^-ATPase. J Biol Chem 272:4795–4803. doi:10.1074/jbc.272.8.4795. 9030535

[25] Lee J, Kim JH, Kim B-N, Kim T, Kim S, Cho B-K, Kim Y-H, Min J. 2020. Identification of novel paraben-binding peptides using phage display. Environ Pollut 267:115479. doi:10.1016/j.envpol.2020.115479. 32892011

[26] Lee S, Lee J, Hwang AR, Kim Y-H, Min J. 2020. A specific nonenal-binding peptide, P4 screened by phage display can remove trans-2-nonenal. Mol Biotechnol 62:273–279. doi:10.1007/s12033-020-00238-y. 32166528

[27] De Plano LM, Carnazza S, Messina GM, Rizzo MG, Marletta G, Guglielmino SP. 2017. Specific and selective probes for Staphylococcus aureus from phage-displayed random peptide libraries. Colloids Surf B Biointerfaces 157:473–480. doi:10.1016/j.colsurfb.2017.05.081. 28654884

[28] Rao SS, Mohan KVK, Gao Y, Atreya CD. 2013. Identification and evaluation of a novel peptide binding to the cell surface of Staphylococcus aureus. Microbiol Res 168:106–112. doi:10.1016/j.micres.2012.07.004. 23017232

[29] Hurst LR, Fratti RA. 2020. Lipid rafts, sphingolipids, and ergosterol in yeast vacuole fusion and maturation. Front Cell Dev Biol 8:539. doi:10.3389/fcell.2020.00539. 32719794 PMC7349313

[30] Nguyen NT, Lee J, Woo SM, Kim YH, Min J. 2021. The response of yeast vacuolar proteins: a novel rapid tool for Salmonella sp. screening. Biotechnol Appl Biochem 68:173–184. doi:10.1002/bab.1910. 32198781

