# Supplementary material for: Targeted Killing of Staphylococcus aureus Using Specific Peptides Displayed on Yeast Vacuoles
Source: Microbiol Spectr. 2023 Apr 26;11(3):e00920-23. doi: 10.1128/spectrum.00920-23 (PMC10269669; doi:10.1128/spectrum.00920-23)
Supplement: Supplemental file 1 — Fig. S1 and S2. Download spectrum.00920-23-s0001.pdf, PDF file, 0.2 MB [file spectrum.00920-23-s0001.pdf]

# Targeted Killing of *Staphylococcus aureus* Using Specific Peptides Displayed on Yeast Vacuoles

**Running title: *S. aureus* Targeted Killing with Yeast Vacuoles and Peptides**

**Jaewoong Lee[1,2#], Ngoc-Tu Nguyen[1,2#], Le-Minh Tran[1],**

**Yang-Hoon Kim[2,3*], and Jiho Min[1*]**

**Supplemental Materials**

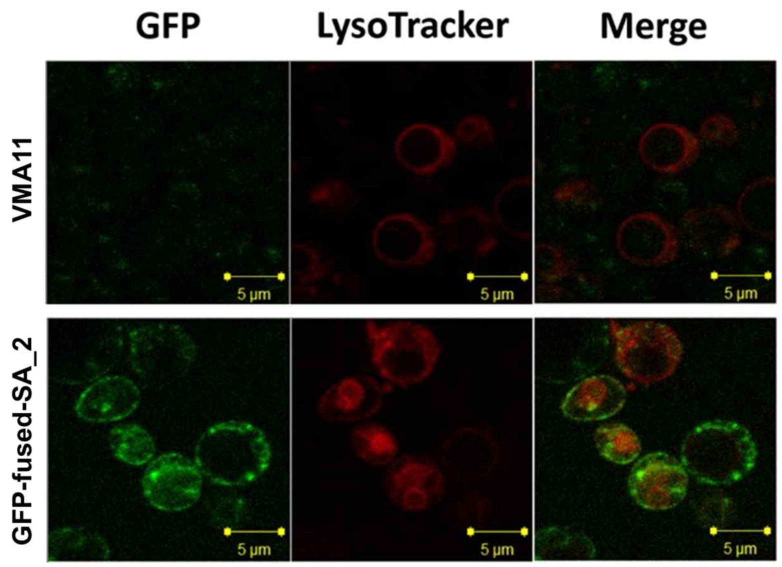


**Figure S1.** GFP-fused-SA_2 was observed to express strongly in the recombinant yeast cell (green signal) and no GFP fluorescence was observed in VMA11 above. Particularly, the green fluorescent signal also merges with the LysoTracker signal (seen as a yellow signal), implying the GFP-fused-SA_2 localization could be on the vacuolar membrane

a)

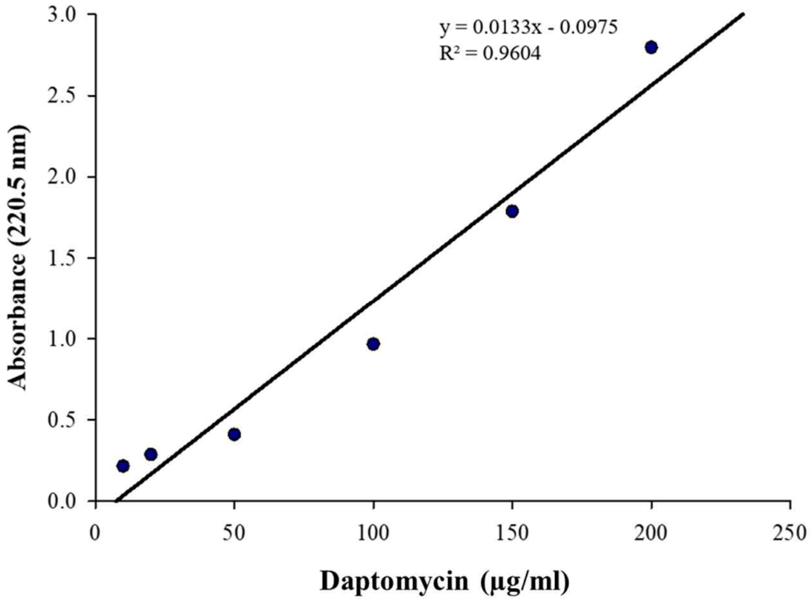


b)

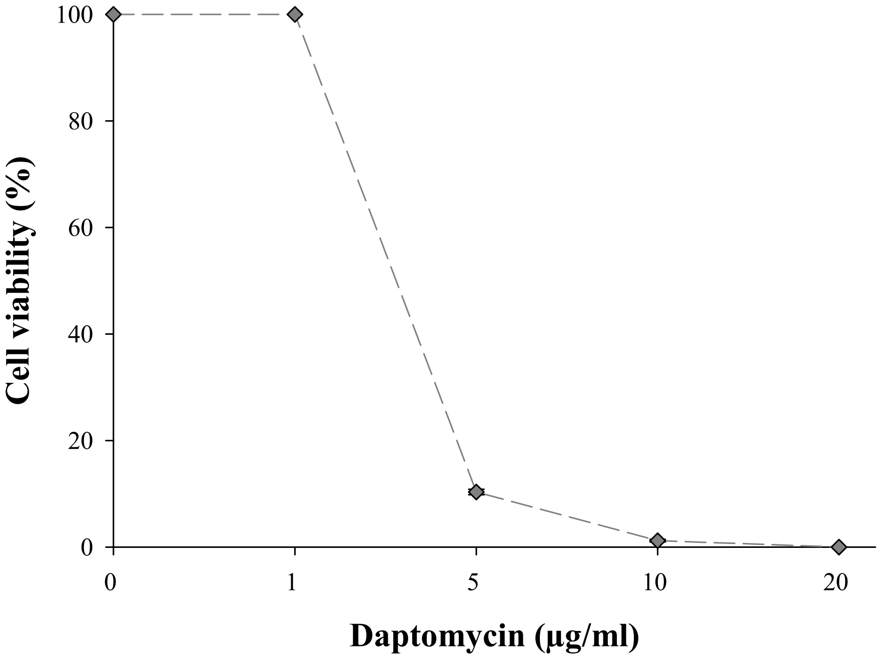


**Figure S2** (a) The standard curve of daptomycin (absorbance at 220.5 nm); (b) Bactericidal effect of daptomycin on *S. aureus*
